# Contribution of genetic variation within SuprMam1 and SuprMam2 to breast cancer susceptibility

**DOI:** 10.1186/1897-4287-10-S2-A90

**Published:** 2012-04-12

**Authors:** M Ratnadiwakara, M Rooke, R Williams, AC Blackburn

**Affiliations:** 1 Molecular Genetics Group, John Curtin School of Medical Research, Australian National University, Canberra ACT 0200, Australia; 2 Molecular System Biology Group, John Curtin School of Medical Research, Australian National University, Canberra ACT 0200, Australia

## 

Two recessive mammary tumour susceptibility loci, *SuprMam1* (for suppressor of mammary tumours) on chromosome 7 (110-140 Mb) and *SuprMam2* on chromosome 2 (120-140MB) have been identified in the BALB/c mouse strain in the Trp53+/- mouse model of spontaneous breast cancer [1]. We studied mammary gland morphology and expression levels of potential candidate genes in SM09 congenic mice (BALB/c *SuprMam* loci in C57BL/6 background) in comparison to parental strains, to identify the genes within the *SuprMam* loci that might be responsible for higher cancer susceptibility in BALB/c mice.

We analysed mammary gland wholemounts for differences in their basic morphology. The average of ductal branch count per 4x field of view was, BALB/c (diestrus) =171, BALB/c (estrus) =173, C57BL/6 (diestrus) =43, C57BL/6 (estrus) =38, SM09 (diestrus) =29 and SM09 (estrus) =23. According to these results, even though there is a significant difference between the numbers of ductal branches between the two parental strains BALB/c and C57BL/6 (estrus:p=0.002, diestrus:p=0.027), there is no significant difference between congenic (SM09) and control (C57BL/6) mice during either estrus (p=0.164) or diestrus (p=0.299) stages at 12 months of age. A similar pattern was observed for total epithelial area. This suggests that BALB/c *SuprMam* alleles alone do not contribute significantly to the morphological differences of the parental strains.

The tumour suppressor DMBT1 (deleted in malignant brain tumors) has previously been identified as a candidate modifier gene within *SuprMam1* (1). Using semi-quantitative RT-PCR, *Dmbt1* mRNA was found to be significantly lower in mammary glands of susceptible BALB/c mice when compared to C57BL/6, while SM09 congenic mice had similar levels to the control C57BL/6 mice. This indicates that the lower level of *Dmbt1* expression in BALB/c mice must be due to transcriptional factor differences outside the region, which is not present in SM09 congenics.

Using Affymetrix data comparing T cell gene expression across 5 different mouse strains, *Cyp2r1*, a major vitamin D hydroxylase, was identified as another potential candidate gene in *SuprMam1*. Plasma 25(OH)D levels were measured in SM09 and control mice to detect the effect of the different *Cyp2r1* alleles. The plasma levels of 25(OH)D were 20% higher in SM09 mice compared to control mice (control= 49.71 nM/L, SM09= 59.44 nM/L). Surprisingly, however, this is the opposite of what is expected based on the association of CYP2R1 expression and cancer susceptibility in human populations.

With these results, we are able to rule out two important candidate genes, DMBT1 and CYP2R1, from being responsible for the higher susceptibility of *SuprMam* loci. In order to confirm these results, and also to identify the possible involvement of other selected candidate genes, we aim to carry out transcriptional profiling on mammary glands of SM09 congenic and control mice.
